# What does anorexia nervosa mean? Qualitative study of the representation of the eating disorder, the role of the family and treatment by maternal caregivers

**DOI:** 10.1192/bjo.2021.27

**Published:** 2021-04-05

**Authors:** Tiziana Marinaci, Luna Carpinelli, Giulia Savarese

**Affiliations:** Department of Medicine, Surgery and Dentistry ‘Scuola Medica Salernitana’, University of Salerno, Baronissi, Salerno, Italy; Department of Medicine, Surgery and Dentistry ‘Scuola Medica Salernitana’, University of Salerno, Baronissi, Salerno, Italy; Department of Medicine, Surgery and Dentistry ‘Scuola Medica Salernitana’, University of Salerno, Baronissi, Salerno, Italy

**Keywords:** Anorexia nervosa, eating disorders, family, maternal caregivers, narrative

## Abstract

**Background:**

Anorexia nervosa is a serious health problem worldwide. The literature widely recognises the roles of the family and caregivers in modulating the onset, development, maintenance and treatment of this disorder. However, few studies have addressed the problem from the perspective of maternal caregivers.

**Aims:**

This study aims to fill this gap by exploring how the meaning given to the term ‘eating disorder’ influences how mothers communicate with each other about a family member's health problems, how they present symptoms and how this problem is managed.

**Method:**

A narrative research project was conducted to capture the mothers’ experiences of living with a daughter diagnosed with anorexia nervosa. In particular, four semi-structured interviews were conducted to explore the ways in which they made sense of the disorder, their roles in treatment and their daughters’ treatment experiences.

**Results:**

The results show that the ways in which mothers characterise the disease guide their method of tackling it and the relationship they have with their daughter, as well as how they see their role in the care and treatment process.

**Conclusions:**

Anorexia is experienced as something that is uncontainable, and a dimension of its accommodation characterises the relationship between mothers and daughters receiving treatment for the disorder. Treatment is accompanied by a delegating dimension, and the clinical implications are discussed in this study.

Caring for a family member, especially a son or daughter with anorexia nervosa, can significantly affect individual family members’ mental and physical health.^1^ The results show that the burden borne by family caregivers who assist people with anorexia nervosa is much more significant than that caused by other psychiatric diseases,^2^ underlining the importance of their involvement in treatment.^3–8^ However, few studies have been concerned with understanding family members’ subjective experiences.^3^ Overall, studies have stressed the importance of considering accounts from the individuals themselves in order to understand their individual experiences and specific needs, and the type of help that can be offered to support them.^3,9,10^ Whitney and colleagues^4^ conducted an interesting study on narratives written by parents. In their analysis, the authors noted that a large part of the discomfort associated with living with anorexia nervosa could be explained by useless assumptions and maladaptive responses to the disease by caregivers. In particular, mothers expressed misperceptions about their own or their daughter's role in causing the disease, or reported an overwhelming emotional response, including sleep deprivation, preoccupation and feelings of hopelessness.

Understanding mothers’ subjective experiences is crucial from at least two points of view. First, as patients’ mothers are usually the ones who are most invested in the treatment and management of the disease, they have a central role in the ways in which patients themselves find meaning in their diagnosis and approach treatment.^11^ From a cultural and social constructionist perspective, the emphasis is here on the idea that the ways in which people experience a disease can be understood as an intimate part of the social system of meaning.^12–14^ In the words of Gergen,^15^ psychopathological categories are ‘socially connoted scripts placed within the sphere of social discourse, which some individuals identify with’ (p. 12). People do not make sense of their experience in isolation but through networks of social interaction, which in turn are rooted in particular social and cultural environments. From this perspective, the ways in which mothers tell the story of their daughter's problems (in terms of where the story starts, which protagonists are involved, which situations are left in the background and which are focused on, and so on), functions connected to patients’ family and care roles (paying attention to behaviours that are considered wrong, praising patients for their efforts, controlling every aspect of the patient's life, asking for expert supervision, asking for help only for the patient or for themselves, and so on) and treatment functions (solving food problems, taking care of the patient's well-being, taking charge of the roles played by the entire family and of the life of the patient, and so on) constitute crucial indicators of the meanings available to patients as they make sense of their disease, its determinants and the care solutions available to them. Second, previous research has highlighted that mothers suffer the greatest emotional burden.^3–5^ Understanding their expectations and their evaluation of treatment can highlight the strengths and limitations that they recognise in the management of anorexia nervosa and may help to detect specific needs that remain unsatisfied in the management of patients by care services.

The present study adopts a qualitative research method to explore mothers’ experience of living with a daughter diagnosed with ongoing anorexia nervosa. In particular, it aims to identify the representations and meanings associated with the disease, the role of the caregiver and the treatment experience.

## Methods

### Instruments

Exploratory in-depth semi-structured interviews were carried out with female caregivers of patients diagnosed with anorexia nervosa on an out-patient basis. The choice of semi-structured interviews was consistent with the aim of exploring the meanings embedded in the mothers’ ways of speaking about the disease, allowing them to touch on any topic that was meaningful to them (Supplementary Appendix 1 available at https://doi.org/10.1192/bjo.2021.27).

The interviews took place by telephone in January 2020 and lasted between 20 and 60 min. Responses to the open-ended questions were recorded in written format by the interviewee. Before each interview, each participant provided informed consent. Descriptive characteristics of patients and mothers, body mass index (BMI), patients’ Eating Disorder Inventory—Third Edition (EDI-3)^16^ scores, and mothers’ McMaster Family Assessment Device (FAD)^17^ scores were also obtained from the medical records provided by the health service where they were recruited. The BMI is a biometric measure based on height and weight. For anorexia nervosa, the DSM-5^18^ requires a significantly lower weight than the normal minimum (i.e. BMI < 18.5). The EDI-3 is a self-report scale that assesses the presence of eating disorder psychopathology and related features. The FAD is a self-report measure that examines seven dimensions of family functioning. The mean score on the general functioning subscale was used in this study. This scale assesses the overall health of the family, with higher scores indicating worse family functioning.

### Sampling and data collection

This study involved mothers caring for daughters with ongoing anorexia nervosa. This intentional choice was consistent with the idea of constructing ‘an opportunity for intensive study’^19^ (p. 446). All patients were managed in an out-patient setting with continuous treatment for at least 1 year in the mental health department of a southern Italian city. All parents participated in a more extensive prospective telemedicine study of therapy and enhanced monitoring of eating disorders.^20^ Via telephone, they were informed about the research goal and asked to participate. The recruited mothers represented 60% of the patients’ female caregivers according to the service. All households were made up of a parental couple and at least two children. As shown in Table 1, the daughters had BMI < 18.5. The EDI-3 scores show how patients 1 and 2 exceeded the cut-off for the clinical diagnosis of anorexia nervosa. In patients 3 and 4, the diagnosis was confirmed by the information obtained from clinical–psychiatric observations and biological indices. The test was administered following repeated hospital admissions, and the scores were not consistent with patients’ perceptions and state of illness. All mothers presented high mean scores on the FAD, highlighting poor family functioning.

**Table 1:** Descriptive characteristics of patients and caregivers

Patient ID	Gender	Age, years	BMI	EDI	Caregiver ID	Gender	Age, years	GF
Patient 1	Female	14	10.85	72	Caregiver 1	Female	45	Mean > 2
Patient 2	Female	16	15.85	72	Caregiver 2	Female	47	Mean > 2
Patient 3	Female	30	16.50	29	Caregiver 3	Female	60	Mean > 2
Patient 4	Female	18	13.84	51	Caregiver 4	Female	44	Mean > 2

BMI, body mass index; EDI, eating disorder inventory; GF, general functioning.

### Data analysis

In this study, the interviews were transcribed verbatim and then analysed qualitatively with the specific purpose of grasping the implicit theories of the interviewees about the topics of the research: representation of the disease (as it can be explained and understood); the role of the family (functions and meanings with respect to the evolution and maintenance of the disorder); and representation of the treatment (roles of health professionals and implicit theories regarding treatment and outcomes). Contrary to analytical techniques, such as content analysis, which emphasises information content, narrative analysis takes on ‘the discontinuity between history and experience and focuses on discourse: on telling about oneself and the devices that individuals use to give meaning to stories’^21^ (p. 162).

We started the analytical work by reading the material in its entirety and listing the topics that the interviewees spoke about. Related themes and content based on the topics of the interviews were then grouped.

### Ethical statement

This study was carried out in accordance with the recommendations of the Associazione Italiana di Psicologia, and all participants gave written informed consent in accordance with the Declaration of Helsinki. As there is no psychological ethics committee at the University of Salerno, ethical review and approval was not required for a study on human participants in accordance with the local legislation and institutional requirements. The protocol was approved by an independent committee from the University's Centro di Counseling Psicologico (Psychological Counselling Centre).

## Results

Three key themes pertaining to the experience of living with and caring for a person with an eating disorder emerged from the analysis of the interviews and are presented below.

### Theme 1: the representation of the disease

#### Anorexia as an uncontainable disorder

Caregivers’ discourse described the disease as something that appeared suddenly within a family context that was often described, until then, as idyllic. The disorder is a set of sudden, uncontainable symptoms or single and discrete events that do not seem to have any anchorage within the family's history and functioning.

‘I don't think that there have been any incidences in the family to cause this, because she has never suffered from anything. Let's say that there has always been a peaceful environment; let's also say that in the family, we have never had great problems.’ (Mother 1)

‘I don't know what the cause is. I don't understand exactly what started this thing.’ (Mother 2)

‘She hardly ever wanted to eat; she only ate vegetables and therefore we asked for advice from a nutritionist. But at the beginning, I did not agree to this; my sister did not agree either, but she gave her advice. She suggested foods to eat and then afterwards she got into the regime of eating balanced foods, but she still could not get out of this thing. This was the start of the symptoms.’ (Mother 2)

‘Because my family was fine and then things happened with her boyfriend. Later on, she broke away a little bit from her sister. There was mourning in the family. Let's say there were various things that happened that led her to be like this, do you understand? Because she was accumulating, accumulating … and therefore she was carrying everything on her shoulders and now she does not have the strength to recover.’ (Mother 3)

‘The depression led her to having anorexia.’ (Mother 3)

‘Then, I don't know if deep inside there is a real cause.’ (Mother 4)

Even the description of the problem and its origin seem to belong to the discourse of other people. The disease was referred to using stories reported by the patients to their mothers or using what care professionals had said:

‘She always says, “maybe I started because I didn't like my legs”. I don't know, she says it was this: she says she started looking at her legs a little more and that she didn't like them and then I don't know. She says that the situation got out of hand; she always says this. Other causes, I don't know, I really don't know … me and her father get on very well; we are not divorced. There has been no mourning in the family. She has always been a pretty, happy girl, at least she says this, but who knows; I don't know.’ (Mother 1)

‘I have no idea, because they say that there is no single underlying cause, that is, in the sense that there can be many things put together. This is what they have told me to date.’ (Mother 4)

Through the mothers’ accounts, a definition of the disease as catalysed by symptoms or individual triggering events emerged. The problem seems to be grafted to a sort of social and relational void that has no roots in the family and that, on the contrary, is related to the uncertainty of events that have happened or may happen from time to time. Within this unpredictability, a sense of anguish and helplessness emerged throughout the narratives of the caregivers. The mothers’ relationships with their daughters, when described, appeared to be fusional: mother and daughter seem to be caught in the same emotional state, which prevents boundaries from being maintained or the problem itself, as well as the patient, from being effectively managed.

‘When this situation started, it was a nightmare, a nightmare, fear. I don't know how I managed to save myself, but we didn't live anymore. Now I'm fine, I'm calm, because she is fine. But before, no. Until she was fine, let's say the whole family was not well, because we quarrelled; there were clashes. It was an unlivable situation. Now that she is well, we are well too. Seeing her happy, seeing her getting ready, seeing her putting on makeup, wanting to go out, it makes me feel comfortable. But when she is not well, I am dying with her, because I am not well either. So now we are well, but there is always the fear of going back to that situation.’ (Mother 2)

In their study on family relations with reference to eating disorders, Leonidas and colleagues^22^ reported on forms of symbiotic relationships that tend to incorporate the other. The authors described these relationships as follows:

‘*A type of relationship between mother and daughter that refers to mutual emotional dependence, in which both members of the pair experience difficulties in differentiating themselves from one another and relate to each other in a more individualized way. In this pattern of codependency, individual borders are blurred. This kind of relationship favours a wide range of conflict, since the pattern of fusional relationship between individuals is inevitably characterized by ambivalence and tension*.’ (p. 1439)

Furthermore, contrary to what would be expected and is widely emphasised in the scientific literature, no mention was made by mothers of their daughters’ bodies or beauty ideals, or about reports involving such dimensions. This aspect is understandable within the context of an idea emerging in the mothers’ narratives that the symptoms and disease develop in a social vacuum and do not need to be explained or related to experiences upon which to reflect. According to Baerveldt and Voestermans,^23^ what is called ‘symptomatic’ also has a social basis. In the case of anorexia nervosa, the body is involved in all kinds of social arrangements. In the mother's account, the decision to leave the body in the background or not mention it becomes a powerful discursive act in itself. Once again, this view assumes that the ways people experience problems are also a reflection of the ‘stories’ they agree to enact.^24^

### Theme 2: the role of family

#### The accommodating family figure

When mothers were asked about the role of the family with respect to the patient's problem and its management, the family was recognised more in terms of accommodating concerning behaviours. The family was acknowledged less in terms of the implications of relationships with the patient and/or the family's role in the onset of the disorder. The family's help was categorised in terms of weight control, nutrition control and the degree of compliance concerning the correct behaviour of the patient.

*‘If there is no help from the family* *… this is something that they need … I am the mother and I can understand her. In fact, I can say that, with her, in the path of care we made together with her … I was more present, because I learned a little bit about how to manage the situation … her sister and her dad, at the beginning, did not understand that it was not necessary to force it, that is, her sister understood it later. However, the family is important … it puts into practice the doctor's advice … this is important because they are with the family at home …’ (Mother 1)*

‘The family can be a resource, but sometimes I realise that it can also be a problem, because in being there, at the limit, sometimes we become too protective as well … that is, I sometimes have difficulties in understanding precisely how to behave with her, whether being too compliant or being too rigid is a bad thing, but the family is certainly a resource.’ (Mother 2)

‘[The family's role] is to just be close to her and pamper her. Sometimes we get angry, sometimes we reassure her when we tell her something that she is doing wrong, for example during dinner and lunch. In any case, my husband always starts, because he says that she shouldn't do it. But I know that this it is not her; if it was her it wouldn't happen, because she is intelligent—she understands. However, she has this mental block that leads her to make mistakes, such as chewing her food and then spitting it out.’ (Mother 3)

‘Eh it is very important, that is, it is fundamental in my opinion, even if I repeat that I don't know how much help we can be. We can help up to a certain point.’ (Mother 4)

The family is a resource by definition, as family members are involved in the patient's daily routine. The dominant responses of the caregivers depict families catalysed by the problem but too busy to reduce the symptoms and to notice the potential role of the family environment in the dynamics of the problem. The desire to reduce the patient's distress appears to be the major motivating factor that drives family accommodation. As a growing number of studies^25–28^ have highlighted, these attempts to reduce distress by caregivers or family members may fuel a cycle of negative reinforcement that increases the level of accommodation. This leads to a greater caregiver burden as well as more severe symptoms, as the patient continues to rely heavily on her accommodating relative(s) and does not develop more independent coping skills.

### Theme 3: the representation of the treatment

#### Treatment as a practice to be delegated

‘[…] but it must be fought above all with psychological talks.’ (Mother 4)

The representation of the disease also influences the expectations of treatment. The disorder is thought of as an enemy to be fought and kept at bay with the help of care professionals, therapeutic interventions and, above all, with good advice put into practice. Maintenance of the patient's relationships and well-being is delegated to the therapist.

‘It went well and she is well also, thanks to the relationships she created with the doctors, so that's okay. Well let's say, for us that meeting was very important, because we didn't even know what to do.’ (Mother 1)

‘I don't know how to behave at times and so I see this as something that is missing from this point of view. That is, I think that we parents also need advice on how to behave with them … often I wanted advice from this point of view and this was missing a little bit.’ (Mother 2)

*‘A mother suffers because she sees her daughter like this. I want to help her with all my heart, but I don't know what to do. This is why we need the psychologist*’*s intervention.’ (Mother 3)*

‘Let's say that as long as she was in Salerno, everything went well for her in her residence; she was followed and everything. I can't say otherwise, just that for me her stay was probably too short. My daughter should have stayed there for longer.’ (Mother 4)

‘Some extra psychotherapy appointments were needed also, because they told me that this is a disorder that should not be treated with drugs … in the residence, every time my daughter or the other girls had a crisis, the psychologist was there to listen to them.’ (Mother 4)

Failure of treatment was thought of as something that starts from the outside: non-incisive treatments, deficient structures and an absence of comparisons with the family concerning the right behaviours to be adopted.

‘I know that from Rome onwards, there are centres where they do more than just one activity, more than one meeting … We only did the meeting with the neuropsychiatrist and the psychologist, then afterwards, we took care of her weight a little. But I know that in other places, other things are done; I don't know in terms of activity, I can't tell you.’ (Mother 1)

‘I see that when she is in therapy, she is a little calmer; on the same day, I already see that she is calmer. But what I missed, in fact I also reported this, was a discussion to see how we parents are … we are left in disarray.’ (Mother 2)

‘My daughter can't stay all this time just hanging on and waiting.’ (Mother 3)

‘They are just short of staff, in my opinion. I mean, a girl in this situation must be seen several times.’ (Mother 4)

The caregivers acknowledged the importance of drug therapy in therapeutic practice but also recognised the necessary coexistence of other mechanisms in the treatment process, such as user listening. This listening, however, is conceived as a specific task of the experts, more than of themselves as caregivers; it is a function of technical competences and persuasive skills.

## Discussion

This study aimed to explore mothers’ experiences of living with a daughter diagnosed with an eating disorder. The qualitative approach allowed us to understand how the individual experiences of mothers can interconnect as shared themes or experience models.^29^

We observed how anorexia nervosa was the central topic addressed by the narratives of all the mothers recruited, highlighting a perspective of the problem as something that belongs to the daughter (more than, for instance, to the functioning and relational dynamics of the family), in which mothers can play a small part in the fight against the disorder. The disorder is experienced as something uncontainable and meaningless, represented as a sudden event that is difficult to manage and/or trace, highlighting the impossibility of recognising how it arises in the first place. In answering the questions ‘Could you tell me about your experience with this problem? What causes do you attribute it to?’ the mothers focused on a set of symptoms, trigger events and failed external relationships. Still, a poor understanding of the deep reasons for their daughter's problems is emphasised, which seems to also be interpretable as a request to help mothers better understand what is happening.

Anorexia nervosa appears like a rupture befalling one out of the blue. It is worth noting two aspects of this way of depicting the problem. First, the ‘rupture’, by definition, points to a deep discontinuity between what happened before – the peaceful experience of the past – and what happened after, the painful and challenging scenario that anorexia nervosa created. Second, as a related aspect, the role of psychological and sociocultural issues in understanding the daughter's anorexia nervosa is downplayed. No discourse emerged concerning specific family or relational dynamics connected with family members, although the literature emphasises treatment constraints and restrictions that may occur owing to the family context. As Le Grange and colleagues^30^ pointed out, ‘eating disorders evolve a multiplicity of family contexts’. This multiplicity reflects different patients’ life stories; the diversity of the relationship models within which each member co-constructs the meaning of their role and their function within the family; and the different resources available to improve mutual living conditions. Accommodation and delegation are the two main mechanisms that emerge from the discourse on the family's role. On the one hand, accommodation emerges as a mechanism that leads to not questioning the particular state of the world in the search for an explanation based on the family dynamics.

According to the conceptual model of family accommodation,^31^ this is a recurrent response on the part of the family when faced with the disorder. ‘Accommodation alleviates distress in the short-term but promotes long-term avoidance and ongoing reliance on accommodation for regulation and coping. These factors contribute to the maintenance and exacerbation of symptoms, in turn causing more distress and ultimately leading to further increases in family accommodation’ (p. 100).

On the other hand, delegation becomes an adaptive response to a situation that one is no longer able to manage. So, the professional's connotation of the professional as the only possible figure who can effectively help to face the problem acquires meaning as a form of ‘soil’ that is now experienced as arid or ‘saturated’.

According to Le Grange and colleagues:^30^

‘[T]he assessment of families requires close attention to the parents’ competencies, motivation, and history of adverse or traumatizing events. But even when such adverse circumstances are present, the development of a plan to help and support sufferers and how to ease family burdens should take precedence over accusation and blame. Thus, in our view, families should be involved routinely in the treatment of most young people with an eating disorder. Exactly how such involvement should be structured, and how it will be most helpful will vary from family to family’ (p. 4).

Overall, the position of an impotent observer of a dramatic problem, which is able to produce anguish with regard to someone else, emerges throughout the interviews.

From a socio-constructive perspective, we emphasise how this feeling of impotence, on the one hand, may affect the way the patients themselves face their problems, and, on the other hand, how it can be nourished by the care context, which can offer a restructuring of shared intelligibility (or not) in order to represent the problem, the goals and the method of care.

Many of the questions and troubles of these mothers appear to be without answers: why did my daughter develop such a problem? What is the aetiology of this problem, which we have learned to call anorexia nervosa? How can we contain the anguish that we have shared? What can I do to wake from this ‘nightmare’? Health services have a crucial role in the elucidation of these answers.

The narratives direct us to a more complex listening space, in which family is more directly involved. This space is not taken for granted: a space where the other is an open field in constant evolution, not known *a priori* or based on maladaptive and rigid aspects. Based on our research considerations, we deem the ‘involvement of narratives’ to be significant in the treatment process. The disorder cannot be considered as a simple manifestation of a maladjusted entity encapsulated in the individual mind; here, it is understood as a by-product of a maladaptive intersubjective and culturally situated process, of which the whole family system is an intrinsic part. This view does not deny the existence of individual vulnerability dimensions that can act in favour of the onset and maintenance of certain maladaptive behaviours. Instead, it underlines the value of psychological aspects (i.e. the meaning) connected to the ways in which the caregivers, patients and actors involved co-construct the sense of their own actions within a specific cultural context of life and relationships. It emphasises the dynamic and field-dependent nature of sense-making.

As Giani^32^ pointed out, ‘in the new context and time of relation, in the hic et Nunc (i.e., the here and now) of the relationship, minds change, and the border between minds becomes specific for those minds – in other words, it becomes an idiosyncratic border – because each of them has changed in the sharing of the time and the place (that is, in the specific relation)’ (p. 174). Consistent with this recognition, Carli^33^ suggested that people seek the help of a psychologist because the psychologist exists, noting that there is no direct link between a certain type of problem and a request for help; rather, this link is mediated by aspects such as the ways the problem (its nature and aetiology), the patient's own role and the role of the expert are represented.

This study has some limitations. First, as recruitment targeted only a few select people, the sample was not indicative of the population of mothers of eating disorder patients. Furthermore, the sample was recruited in the specific context of patients undergoing intervention. As such, the sample may reflect the specific culture of the service (e.g. its way of promoting a specific view of the problem and the treatment). On the other hand, the study did not aim to be representative of mothers’ narratives concerning anorexia nervosa; instead, it aimed to capture aspects of the discourse to highlight how mothers consider the problem and what expectations they bring to the treatment process. Further research should be carried out in order to explore the (dis)similarities of the stories that can emerge within different family, social and care contexts.

## Data Availability

Anonymised data are available upon request.

## Supplementary material

For supplementary material accompanying this paper visit http://dx.doi.org/10.1192/bjo.2021.27. click here to view supplementary material
