# Supplementary material for: What does anorexia nervosa mean? Qualitative study of the representation of the eating disorder, the role of the family and treatment by maternal caregivers
Source: BJPsych Open. 2021 Apr 5;7(3):e75. doi: 10.1192/bjo.2021.27 (PMC8086394; doi:10.1192/bjo.2021.27)
Supplement: Supplementary file 1 [file S2056472421000272sup001.zip › Appendix_1.docx]

Appendix 1

Semi-structured interviews

1. Could you tell me about your experience with this problem? What causes do you attribute to it?
2. What role do you see your family having with respect to this problem and its management?
3. Could you tell me about your experience with the treatment that your family member has been receiving?
4. According to your point of view and experience, what objectives should the service pursue in order to improve the path of patient care?
